# Supplementary material for: COVID-19 gender susceptibility and outcomes: A systematic review
Source: PLoS One. 2020 Nov 3;15(11):e0241827. doi: 10.1371/journal.pone.0241827 (PMC7608911; doi:10.1371/journal.pone.0241827)
Supplement: S1 File — (DOCX) [file pone.0241827.s002.docx]

Supplementary Material

1. **Search strategy**

A search of Medline via PUBMED interface, Web of Science and the Cochrane Library was conducted using the following search terms: “2019 novel coronavirus” OR “SARS-CoV-2” OR “2019-nCoV” OR “novel coronavirus”. We then restricted the search to articles on adult humans. In the last stage of the database search, articles not written in English and those conducted on animal models and special populations (children, pregnant women) were also excluded ((((((("severe acute respiratory syndrome coronavirus 2"[Supplementary Concept] OR "severe acute respiratory syndrome coronavirus 2"[All Fields]) OR "2019 novel coronavirus"[All Fields]) OR (("severe acute respiratory syndrome coronavirus 2"[Supplementary Concept] OR "severe acute respiratory syndrome coronavirus 2"[All Fields]) OR "sars cov 2"[All Fields])) OR (("severe acute respiratory syndrome coronavirus 2"[Supplementary Concept] OR "severe acute respiratory syndrome coronavirus 2"[All Fields]) OR "2019 ncov"[All Fields])) OR ((("novel"[All Fields] OR "novel s"[All Fields]) OR "novels"[All Fields]) AND (("coronavirus"[MeSH Terms] OR "coronavirus"[All Fields]) OR "coronaviruses"[All Fields]))) AND "humans"[MeSH Terms]) Filters: English, MEDLINE, Adult: 19+ years). The search was conducted twice, the first time on May 12, 2020, and the second one on June 1, 2020 (date of last access to the databases to search for studies).

1. **Funnel plots**

**Figure S1: Funnel plots for each outcome**

**a) Mortality**

**b) Severity**

**c) ICU admission**

OR: Odds ratio

SE(log OR): standard error of the effect estimate

1. **Sensitivity analysis**

1. **Newcastle Ottawa scale modified and adapted to the purpose of our study**

| **Assessment of quality of a cohort study – Newcastle Ottawa Scale** |  |
| --- | --- |
| **Selection** (tick one box in each section) |  |
| 1. Representativeness of the intervention cohorta) truly representative of the average hospitalized COVID-19 patient: PCR+, symptomaticb) somewhat representative of the average hospitalized COVID-19 patient: PCR+, symptomaticc) selected group of patients, e.g. only certain pathologies (diabetes) or categories of admission or a specific recruitment (eg only ICU)d) no description of the derivation of the cohort | 🞏★  🞏★  🞏  🞏 |
|  |  |
| 2. Ascertainment of severitya) Reviewed with a clear definitionc) No clear definition of severityd) other / no description | 🞏★  🞏  🞏 |
|  |  |
| **Comparability (tick one or both boxes, as appropriate**) |  |
| 1. Comparability of cohorts on the basis of the design or analysisa) study controls for gendered age, hospitalization, ICU, severity, mortalityb) study controls for gendered additional factors TTT | 🞏★  🞏★ |
| **Outcome** (tick one box in each section) |  |
| 1. Assessment of outcomea) prospectively recorded b) chart review or database records c) self-report d) other / no description | 🞏★  🞏★  🞏 |
| 2. Was follow up long enough for outcomes to occura) yes, if median duration of follow-up >= 28 days or ICU discharge or deathb) no, if median duration of follow-up < 28 days or ICU discharge or death | 🞏★  🞏 |
| 3. Adequacy of follow up of cohortsa) complete follow up: all subjects accounted forb) subjects lost to follow up unlikely to introduce bias: number lost <= 10%,  or description of those lost suggesting no different from those followedc) follow up rate < 90% and no description of those lostd) no statement | 🞏★  🞏★  🞏  🞏 |
